# Changes in Corneal Asphericity after MyoRing Implantation in Moderate and Severe Keratoconus

**DOI:** 10.18502/jovr.v14i4.5443

**Published:** 2019-10-24

**Authors:** Masoud Khorrami-Nejad, Ozra Aghili, Hesam Hashemian, Mohamad Aghazadeh-Amiri, Farshid Karimi

**Affiliations:** ^1^Department of Optometry, School of Rehabilitation, Shahid Beheshti University of Medical Sciences, Tehran, Iran; ^2^Eye Research center, Farabi Eye Hospital, Tehran University of Medical Sciences, Tehran, Iran

**Keywords:** Cornea, Corneal Topography, Keratoconus

## Abstract

**Purpose:**

To evaluate the effect of MyoRing implantation on corneal asphericity in moderate and severe keratoconus (KCN).

**Methods:**

This cross-sectional observational study comprised 32 eyes of 28 patients with KCN, who had femtosecond-assisted MyoRing corneal implantation. The primary outcome measures were preoperative and six-month postoperative corneal asphericity in 6-, 7-, 8-, 9-, and 10-mm optical zones in the superior, inferior, nasal, temporal, and central areas. The secondary outcome measures included uncorrected distance visual acuity (UDVA), corrected distance visual acuity (CDVA), manifest refraction, thinnest location value, and keratometry readings.

**Results:**

A significant improvement in the UDVA and CDVA was observed six months after the surgery (*P* < 0.001) with a significant reduction in the spherical (4.67 diopters (D)) and cylindrical (2.19 D) refractive errors. A significant reduction in the corneal asphericity in all the optical zones and in the superior, inferior, nasal, temporal, and central areas was noted (*P* < 0.001). The mean thickness at the thinnest location of the cornea decreased from 437.15 ± 30.69 to 422.81 ± 36.91 μm. A significant corneal flattening was seen. The K1, K2, and Km changes were 5.32 D, 7 D, and 6.17 D, respectively (*P* < 0.001).

**Conclusion:**

MyoRing implantation is effective for improving corneal asphericity in patients with KCN. It allows successful corneal remodeling and provides a significant improvement in UDVA, CDVA, and refractive errors.

##  Introduction

Keratoconus (KCN) is the most prevalent primary corneal ectasia which commonly causes bilateral asymmetrical thinning of the paracentral cornea.^[1]^ Thinning of the cornea usually occurs in the central and the inferior temporal region,^[2]^ although thinning in the upper area has also been reported.^[3,4]^ Thinning of the cornea leads to myopia and irregular astigmatism, which affects visual quality.

In most studies, the reported prevalence of KCN in the general population was between 50 and 230 per 100,000.^[5,6,7]^ Recent studies have shown that KCN starts from the posterior corneal surface and the posterior curvature is affected to a greater extent than the anterior curvature in the early stage of KCN.^[8,9]^ Therefore, devices based on direct evaluation of the two surfaces of the cornea (Scheimpflug principle) have been specifically addressed in recent studies.^[10,11]^ Corneal topography, the most accurate method for detecting and confirming KCN, is also based on Placido disc and Scheimpflug imaging, which are sensitive methods for assessing the shape of the cornea.^[12,13]^

Since the cornea accounts for the largest amount of the dioptric power of the eye, a small change in the shape of the cornea is sufficient to produce a significant dioptric change.^[14]^ Aspheric cornea affects the lower and the higher order aberrations, particularly compensates for the spherical aberrations, and ultimately improves the retinal image quality. Therefore, corneal asphericity has a potentially significant effect on visual quality.^[15,16]^

MyoRing is a 360° continuous full-ring implanted into a corneal pocket using a PocketMaker microkeratome or a femtosecond laser. The MyoRing has a diameter of 5–8 mm, a thickness of 200–400 μm, and a body width of 0.5 mm. MyoRing implantation has proven to be safe and effective for the treatment of myopia, ectasia, and KCN.^[17,18,19]^ The use of an intrastromal corneal ring in the periphery of the cornea causes frontal displacement of the cornea. It also causes central corneal flattening (the arc-shortening effect).^[20]^

Very few studies have evaluated the corneal asphericity before and after MyoRing implantation. Due to the effect of corneal asphericity on the quality of vision, we aimed to study the changes in these parameters in our study. The prediction of the effect of MyoRing on corneal asphericity may determine whether the patient is a good candidate for MyoRing implantation. The selection of suitable candidates for this surgery may increase the quality of vision and, ultimately, the quality of life.

##  METHODS

This cross-sectional observational study included 32 eyes of 28 patients with a mean age of 26.75 ± 6.46 years (ranging 18 to 42 years).

The statistical population of this study consisted of stage 2 and stage 3 KCN patients referred to the Farabi Eye Hospital between 2014 and 2016 who underwent femtosecond-assisted MyoRing implantation. Thirty-two eyes of 28 patients were included in the study and all of these 28 patients were evaluated. This study was performed in accordance with the tenets of the Declaration of Helsinki, and the Ethics Committee of the Shahid Beheshti University of Medical Sciences approved the study. The records were de-identified and anonymous.

The exclusion criteria for this study consisted of any defect in documentation, presence of corneal scar, previous corneal surgery of any type, use of contact lenses three weeks before surgery, presence of other ocular or systemic diseases (retinal abnormalities, diabetic retinopathy, cataract, glaucoma, etc.), the inability of acquiring reliable imaging, and failure to complete postoperative examinations six months after the procedure. The records of the patients included in the study were examined by the observation method.

In all cases, the pocket for MyoRing implantation was created using the 150 kHz femtosecond technology (IntraLase, Advanced Medical Optics Inc., Santa Ana, CA, USA). After the creation of the pocket, a space was gently formed by passing a spatula through the temporal incision between the closed flap and the bed.

Data extracted from the patients' records included the axes and amount of total, anterior, and posterior corneal astigmatism measured by the Pentacam (Oculus Optikger te GmbH, Wetzlar, Germany), as well as the spherical and cylindrical refractive errors. Corneal asphericity in different zones of the cornea was measured using a refractive map of the Pentacam. All patients fulfilling the inclusion criteria during the study period were included in the study. All surgeries were performed by a single surgeon.

In the present study, MyoRings with diameters of 5 mm and 6 mm and thicknesses of 240 μm and 280 μm were used according to the size of the pupil in mesopic conditions and the severity of KCN. Uncorrected distance visual acuity (UDVA), corrected distance visual acuity (CDVA), manifest refraction, corneal asphericity at 6, 7, 8, 9, and 10 mm optical zones in the superior, inferior, nasal, temporal, and central areas, minimum corneal thickness, and keratometry readings were the outcome measures of the study. They were measured preoperatively and six months after MyoRing implantation.

KCN patients were divided into four categories according to the Amsler Krumeich KCN classification.^[21]^ The stage was determined if one characteristic each from the following was present:

Grade 1: induced myopia and/or astigmatism less than 5 D, keratometric reading ≤ 48.00 D, Vogt's striae, and/or eccentric steepening; grade 2: induced myopia and/or astigmatism of 5–8 D, keratometric reading less than 53 D, lack of scar, and/or minimum corneal thickness greater than 400 μm; grade 3: induced myopia and/or astigmatism of 8–10 D, mean keratometric reading greater than 53 D, no scar, and/or minimum corneal thickness of 200–400 μm; and grade 4: refraction not measurable, the keratometric reading greater than 55 D, central scar, and/or the corneal thickness less than 200 μm.

In the next step, patients with grade 2 and grade 3 KCN who underwent the MyoRing implantation and fulfilled the inclusion criteria for the study were selected as the statistical population. Data were gathered using Pentacam HR (Oculus Optikger te GmbH, Wetzlar, Germany), Topcon auto-kerato refractometer KR 8800 (Topcon Corporation, Tokyo, Japan), Heine Beta 200 retinoscope (Heine Optotechnik GmbH & Co., Herrsching, Germany), and an ETDRS chart (at a distance of 4 m). Visual acuity was recorded using the LogMAR system.

Statistical analysis was performed using the SPSS software for Windows (Version 22, IBM Inc., Armonk, New York, USA). Data were analyzed using statistical indices, including the mean and standard deviation. All data were checked for normality using the Shapiro–Wilk test, followed by a comparison of the data before surgery with those after six months of surgery using the paired *t*-test. To compare the normal variables in multiple groups, one-way or two-way analysis of variance was used. *P* < 0.05 was considered statistically significant.

##  RESULTS

In the present study, 32 eyes of 28 patients [13 (40.6%) males and 19 (59.4%) females] with stage 2 and stage 3 of KCN were examined. The mean age was 26.75 years, with a range of 18 to 42 years. Six-month postoperative follow-up was carried out for all patients. Eighteen right eyes (25.56%) and fourteen left eyes (75.43%) were included in the study. According to the Amsler Krumeich KCN classification, 24 eyes (75%) had grade 2 KCN and 8 eyes (25%) had grade 3 KCN. The mean spherical and cylindrical refractive errors and the mean LogMAR CDVA and UDVA before and after the MyoRing implantation are presented in Table 1. The mean spherical and cylindrical refractive errors significantly decreased (*P* < 0.001) and the mean UDVA and CDVA improved significantly after the surgery (*P* < 0.001).

**Table 1:** Descriptive statistics for refractive errors, the corrected distance visual acuity, and the uncorrected distance visual acuity


**Variables**		**Unit**	**Mean**	**Maximum**	**Minimum**	**Standard deviation**	**** ***P*** **-value**
Sphere	Pre-operation	Diopter	–4.95	0	–11.00	3.03	< 0.001
	Post-operation	–2.08	+4.00	–4.00	1.60	
Cylinder	Pre-operation	Diopter	–4.28	–1.00	–8.00	1.85	< 0.001
	Post-operation	–2.08	0	–6.00	1.32	
CDVA	Pre-operation	LogMAR	0.32	0.1	0.5	0.17	< 0.001
	Post-operation	0.22	0	0.5	0.15	
UDVA	Pre-operation	LogMAR	1.12	0.4	2.2	0.45	< 0.001
	Post-operation	0.44	0.1	1.6	0.31	
	
	
CDV, corrected distance visual acuity; logMAR, logarithm of the minimum angle of resolution; UDVA, uncorrected distance visual acuity							

Means and standard deviations of minimum, average, and maximum keratometry before the surgery were 48.86 ± 3.14, 51.19 ± 3.6, and 53.63 ± 54.3 D, respectively. The mean and standard deviation of minimum, average, and maximum keratometry six months after the MyoRing implantation were 43.34 ± 3.36, 45.01 ± 36.36, and 46.59 ± 3.67 D, respectively, whereas those for the thickness at the thinnest point of the cornea were 437.15 ± 30.69 μm before the surgery and 422.181 ± 36.91 μm after the MyoRing implantation [Table 2]. The thinnest point of the cornea after the surgery was 14.34 ± 16.53 μm thinner than the preoperative value (*P* < 0.001).

**Table 2:** Descriptive statistics of the keratometry and the thinnest point of the cornea


**Variables**		**Unit**	**Mean**	**Minimum**	**Maximum**	**Standard deviation**	**P-value**
Thinnest point	Pre-operation	μm	437.15	380	486	30.69	< 0.001
	Post-operation	422.81	343	491	36.91	
K-Min	Pre-operation	Diopter	48.86	43.3	54.1	3.14	< 0.001
	Post-operation	43.54	36.8	50.3	3.36	
K-Max	Pre-operation	Diopter	53.69	45.3	60.3	3.54	< 0.001
	Post-operation	46.59	38.2	54.8	3.67	
K-Mean	Pre-operation	Diopter	51.19	44.3	56.7	3.06	< 0.001
	Post-operation	45.01	38	51.4	3.36	
	
	
K-Max, maximum keratometry values; K-Mean, mean keratometry values; K-Min, minimum keratometry values							

The means and the standard deviations of the asphericity based on the position of the cornea (nasal, temporal, upper, and lower) in the 6-, 7-, 8-, 9-, and 10-mm optical zones are presented in Table 3. The mean and standard deviation of the asphericity in the entire cornea were also determined for these optical zones [Table 3]. The mean corneal asphericity before the surgery was the highest in the 6-mm zone and the lowest in the 10-mm zone. However, after the MyoRing implantation, the greatest and the least changes in the corneal asphericity values were observed in the 6-mm and 10-mm zones, respectively.

**Table 3:** Descriptive statistics of the asphericity variables based on the corneal zones


**Asphericity**	**Zone**	**Preoperation**		**Postoperation**	
	**Mean**	**Standard deviation**	**Mean**	**Standard deviation**
Nasal	6 mm	–2.14	1.3	0.6	1.23
	7 mm	–1.72	0.88	0.26	0.92
	8 mm	–1.51	0.63	–0.07	0.73
	9 mm	–1.38	0.49	–0.38	0.62
	10 mm	–1.21	0.44	–0.55	0.55
Temporal	6 mm	–0.97	0.72	1.11	1.29
	7 mm	–0.93	0.57	0.7	0.99
	8 mm	–0.92	0.42	0.33	0.78
	9 mm	–0.90	0.32	0.01	0.61
	10 mm	–0.87	0.34	–0.25	0.46
Superior	6 mm	–1.86	1.2	0.28	1.58
	7 mm	–1.69	0.83	0.39	1.2
	8 mm	–1.47	0.61	0.29	0.89
	9 mm	–1.26	0.48	0.05	0.69
	10 mm	–1.09	0.4	–0.21	0.51
Inferior	6 mm	–0.10	0.94	1.51	1.9
	7 mm	–0.43	0.73	0.92	1.48
	8 mm	–0.66	0.64	0.44	1.18
	9 mm	–0.85	0.56	0.06	0.96
	10 mm	–0.97	0.49	–0.27	0.78
Entire cornea	6 mm	–1.27	0.64	0.88	1.19
	7 mm	–1.20	0.51	0.57	0.94
	8 mm	–1.14	0.4	0.25	0.73
	9 mm	–1.07	0.34	–0.06	0.57
	10 mm	–0.97	0.45	–0.29	0.46
	
	

The change in the mean corneal asphericity was significant in all optical zones after the MyoRing implantation (*P* < 0.001). The changes in the mean temporal, nasal, upper, and lower asphericity in all optical zones of the cornea after the surgery were also found to be significant (*P* < 0.001).

##  DISCUSSION

The radius of curvature of a cornea with KCN changes after the MyoRing implantation, making it flatter. Due to this change in shape, the cornea with KCN may become similar to a normal physiological cornea after the surgery. However, creating a flattening effect in the center of the cornea without following a specific asphericity pattern leads to a positive asphericity (rather than improvement in the negative diopter value) of the cornea and the cornea may become completely oblate after surgery. To achieve excellent results using intrastromal rings, it is necessary to control the flattening pattern induced by them.

In the present study, significant decreases in myopia and the cylindrical refractive errors were observed, with a greater reduction in myopia than in astigmatism at six months postoperatively. The mean reductions in the spherical and cylindrical refractive errors were 4.67 and 2.19 D, respectively. These findings are consistent with those of previous studies. In the study by Daxer et al, the mean variations in the spherical and cylindrical refractive errors were 5.23 and 2.23 D, respectively, which are comparable with our results.^[22]^ In another study by Jabbarvand et al, the average postoperative changes in the spherical and cylindrical refractive errors were 5.74 and 3.23 D, respectively.^[23]^ The follow-up evaluations were performed 1, 6, and 12 months after the surgery, with similar results at each evaluation.

The amount of myopia and astigmatism correction achieved in our study was greater than that in the studies using intracorneal ring segment (ICRS) implantation for the treatment of KCN.^[24,25]^ Therefore, MyoRing may have more potential for the correction of myopia and astigmatism than ICRS in patients with KCN. This is probably due to the arc-shortening effect of complete rings.

In the present study, the reduction in the spherical and cylindrical refractive errors after MyoRing implantation was consistent with a significant improvement in visual acuity, as expected. The mean increase in the UDVA after the surgery was 0.68 ± 0.44 LogMAR, a finding similar to those from the studies by Jabarvand et al^[23]^ and Alio et al.^[19]^ Improvement in the UDVA by 0.62 and 0.75 LogMAR, respectively, has been reported in both the studies.

In the present study, the mean improvement in the CDVA was 0.1 LogMAR, which is similar to the findings in the study by Alio et al.^[19]^ In other studies by Mahmood et al^[26]^ and Jabarvand et al,^[23]^ the mean improvement in the CDVA was 0.17 LogMAR and 0.26 LogMAR, respectively. In a study conducted by Saeed et al, no significant change was observed in the thickness of the thinnest point of the cornea after the MyoRing implantation.^[27]^ However, in the present study, a statistically significant decrease of 14.34 μm in the thickness of the thinnest point of the cornea was observed.

In the present study, a significant central flattening of the corneal topography was observed six months after the surgery, which was consistent with the refractive changes. Based on the results obtained in this study, the mean keratometry was reduced by 6.17 D, which is similar to the result of an average keratometric reduction by 5.76 diopters in the study by Daxer et al.^[22]^ In the studies by Alio et al^[19]^ and Mahmood et al,^[26]^ this decrease was approximately 8 D, which is slightly higher than that found in our study. In the study by Alio et al, the pupil size was not considered in the mesopic conditions, and only the MyoRings with a diameter of 5 mm and a thickness of 280 μm were implanted in all patients. In the present study, based on the size of the pupil of the patients under the mesopic conditions and on preoperative keratometry, MyoRings with diameters of 5 and 6 mm and thicknesses of 240 and 280 μm were implanted. The smaller diameter and higher thickness of the MyoRing increase the effect of corneal flattening by the ring (the arc-shortening effect).^[23]^ The reason for the difference in the keratometric reduction between the two studies may be the difference in the size and thickness of the MyoRing. In the study by Mahmood et al, the size of the pupil in patients under mesopic conditions was ignored as the sample size was small (six eyes). Two patients had a cross-linking history. Moreover, the study did not take into account the specific inclusion and exclusion criteria.

Jabbarvand et al reported a 9.78 D reduction in the mean keratometry reading,^[23]^ which is significantly different from that in the present study and in other studies. The authors used an Orbscan to evaluate the anterior corneal surface, while in the present study, we used the Pentacam HR for corneal imaging. According to some studies, the reproducibility of Pentacam is higher than that of Orbscan.^[28]^ In the study by Jabbarvand et al, 70% of the patients were male, and the minimum age of the subjects was 19 years with no maximum age limit. In the present study, approximately 60% of the patients were female and the age range was 18–42 years.

We observed a significant change in the corneal asphericity after the MyoRing implantation. After the surgery, the corneal prolate shape was reduced and the cornea became oblate. The result was predictable because a considerable flattening was achieved in the center of the cornea with the MyoRing implantation. An interesting finding was that the mean decrease in the asphericity at the 6–10-mm zones followed the similar slope.

In this study, the nasal position of the cornea had the highest asphericity changes, and the inferior of the cornea had the lowest amount of asphericity compare to other regions (In the 6-mm optical zone).

It is our belief that the present study was the first study that evaluated the changes in corneal asphericity in the 6–10-mm optical zones according to the nasal, temporal, upper, and lower corneal regions after MyoRing implantation. Alio et al studied the changes in the mean corneal asphericity in the 8-mm zone.^[19]^ They reported a decrease of 1.72 in the mean asphericity six months after the surgery, which is almost consistent with the present study (1.39 ± 0.56). A slight difference between the two studies may be due to a greater corneal flattening in the study by Alio et al due to the use of MyoRings with a smaller diameter and a greater thickness. In their study, asphericity changes were evaluated one and two months postoperatively. This follow-up period is shorter than the follow-up of six months in the present study.

In another study by Hosny et al, the corneal asphericity was evaluated only in a MyoRing with a diameter of 6 mm.^[29]^ The mean change in the corneal asphericity was 0.43 ± 2.6 after the MyoRing implantation. The less favorable outcome of this study compared to the present study could be due to the difference in the duration of follow-up after the surgery. In the study by Hosny et al, the results were evaluated one month after the surgery.

Torquetti et al evaluated the changes in the corneal asphericity after implantation with ICRSs in KCN.^[16]^ They reported a mean decrease of 0.53 in the corneal asphericity. The mean asphericity before the surgery in their study was –0.85. They suggested that the change in the thicker and the paired segments was more than in the single segments. The MyoRing has a stronger arc-shortening effect than the ICRSs due to its 360° design. The reason for the low amount of variation in the asphericity in the study by Torquetti et al may be the use of ICRSs instead of the MyoRing.

In conclusion, we found that MyoRing implantation significantly reduced the cylindrical and spherical refractive errors due to central corneal flattening. This technique appears to be effective for decreasing myopia and corneal steepness, and effectively affects the corneal shape. A more detailed examination of the corneal parameters such as asphericity in patients with KCN can result in better understanding of the changes associated with KCN. Positive spherical aberration worsens contrast sensitivity and may compromise the visual outcomes after MyoRing implantation.

##  Financial Support and Sponsorship

Nil.

##  Conflicts of Interest

There are no conflicts of interest.
